# Shikonin suppresses ERK 1/2 phosphorylation during the early stages of adipocyte differentiation in 3T3-L1 cells

**DOI:** 10.1186/1472-6882-13-207

**Published:** 2013-08-06

**Authors:** So Young Gwon, Ji Yun Ahn, Chang Hwa Jung, Bo Kyung Moon, Tae Youl Ha

**Affiliations:** 1 Functional Food Technology Research Group, Korea Food Research Institute, 516, Baekhyn-dong, Seongnam-si, Gyeonggi-do 463-746, Republic of Korea; 2 Department of Food and Nutrition, Chung-ang University, Anseong-si, Republic of Korea

**Keywords:** Shikonin, ERK 1/2, 3T3-L1 adipocyte, Adipogenesis, Anti-obesity

## Abstract

**Background:**

The naphthoquinone pigment, shikonin, is a major component of *Lithospermum erythrorhizon* and has been shown to have various biological functions, including antimicrobial, anti-inflammatory, and antitumor effects. In this study, we investigated the effect of shikonin on adipocyte differentiation and its mechanism of action in 3T3-L1 cells.

**Methods:**

To investigate the effects of shikonin on adipocyte differentiation, 3T3-L1 cells were induced to differentiate using 3-isobutyl-1-methylzanthine, dexamethasone, and insulin (MDI) for 8 days in the presence of 0–2 μM shikonin. Oil Red O staining was performed to determine the lipid accumulation in 3T3-L1 cells. To elucidate the anti-adipogenic mechanism of shikonin, adipogenic transcription factors, the phosphorylation levels of ERK, and adipogenic gene expression were analyzed by Western blotting and quantitative real-time PCR. To further confirm that shikonin inhibits adipogenic differentiation through downregulation of ERK 1/2 activity, 3T3-L1 cells were treated with shikonin in the presence of FGF-2, an activator, or PD98059, an inhibitor, of the ERK1/2 signaling pathway.

**Results:**

Shikonin effectively suppressed adipogenesis and downregulated the protein levels of 2 major transcription factors, PPARγ and C/EBPα, as well as the adipocyte specific gene aP2 in a dose-dependent manner. qRT-PCR analysis revealed that shikonin inhibited mRNA expression of adipogenesis-related genes, such as PPARγ, C/EBPα, and aP2. Adipocyte differentiation was mediated by ERK 1/2 phosphorylation, which was confirmed by pretreatment with PD98059 (an ERK 1/2 inhibitor) or FGF-2 (an ERK 1/2 activator). The phosphorylation of ERK1/2 during the early stages of adipogenesis in 3T3-L1 cells was inhibited by shikonin. We also confirmed that FGF-2-stimulated ERK 1/2 activity was attenuated by shikonin.

**Conclusions:**

These results demonstrate that shikonin inhibits adipogenic differentiation via suppression of the ERK signaling pathway during the early stages of adipogenesis.

## Background

Obesity can be defined as increased fat mass due to increases in the number and size of adipocytes [1]. Adipose tissue plays an important role in lipid metabolism, including the storage of triglycerides and fatty acid release. Adipocytes secrete numerous adipokines, including leptin, adiponectin, and resistin. Therefore, white adipose tissue is crucial for the maintenance of energy homeostasis and highly influences obesity.

Adipogenesis involves undifferentiated preadipocytes converting to differentiated adipocytes and plays a key role in fat mass growth [2,3]. Controlling adipogenesis is a potential strategy for obesity prevention [4]. Many studies have demonstrated that natural compounds, such as quercertin, genistein, and esculetin, inhibit adipogenesis [5-7]. Adipogenesis is regulated by a number of transcription factors, such as CCAAT/enhancer binding proteins (C/EBPs) and peroxisome proliferator-activated receptor γ (PPARγ). C/EBP β and C/EBP δ rapidly induces the expression of PPARγ and C/EBPα. PPARγ and C/EBPα activate the expression of a number of genes induced during adipocyte differentiation, including genes responsible for lipid accumulation and insulin sensitivity [8]. The mitogen-activated protein kinase (MAPK) pathway regulates the expression of adipogenic transcription factors during the adipogenesis [9]. MAPKs comprise three groups: extracellular signal-regulated kinases 1 and 2 (ERK1/2), c-Jun amino-terminal kinases (JNKs), and p38. The extracellular signal-regulated kinases 1 and 2 (ERK1/2) regulate cell proliferation and are necessary for initiating the differentiation process in pre-adipocyte [10]. For example, ERK phosphorylation was increased during the early stages of adipocyte differentiation in embryonic stem cells [11].

Shikonin, the major compound in *Lithospermum erythrorhizon* (LE), has many beneficial effects on wound healing, including anti-inflammatory and anti-tumor effects [12-14]. Recent studies have shown that shikonin derivatives inhibit adipogenesis [15,16]. Our previous study demonstrated the active compounds of *L. erythrorhizon*; acetylshikonin has been shown to exert anti-obesity effects in vivo [17]. Yoon et al. also demonstrated the anti-adipogenic functions of shikonin in adipocyte differentiation [15,16]. Based on these reports, we explored the antiobesity effect of shikonin as a potential ERK inhibitor. In addition, the effects of shikonin on 3T3-L1 cells at early differentiation stages have not been reported. Therefore, this study sought to characterize the effects of shikonin, focusing on ERK phosphorylation during the early stages of adipogenesis in 3T3-L1 cells, and explored possible underlying molecular mechanisms.

## Methods

### Cell culture and differentiation

3T3-L1 mouse fibroblast cells (American Type Culture Collection Manassas, VA, USA) were cultured in Dulbecco’s modified Eagle’s medium (DMEM) containing 10% calf serum, 100 U/ml penicillin, 100 μg/ml streptomycin, and 2 mM L-glutamine (Invitrogen, Carlsbad, CA, USA) at 37°C under 5% CO_2_. On day 3 after confluence (day 0), the cells were exposed to differentiation medium (DMEM containing 0.25 mM 3-isobutyl-1-methylzanthine, 0.25 μM dexamethasone, and 1 μg/mL insulin [MDI] with 10% FBS) for 2 days. The cells were cultured for another 2 days in DMEM containing 1 μg/mL insulin and 10% FBS. The cells were then maintained in postdifferentiation medium (DMEM containing 10% FBS), and the medium was replaced every 2 days. To evaluate the effects of shikonin on preadipocyte differentiation, the cells were cultured with differentiation medium in the presence of various concentrations (0.5-2 μM) of shikonin. Shikonin was purchased from Calbiochem (San Diego, CA, USA). A range of concentrations of shikonin was prepared by serial dilution of a stock solution with DMSO. The cells were harvested on day 8, when differentiation was complete. For early-stage adipogenesis analysis, the cells were treated with PD98059 or FGF-2 and harvested hourly.

### MTT assay

Cell viability was determined by an MTT assay in 96-well plates. Pre-adipocytes were seeded at a density of 5 × 10^3^ cells per well. After 24 h incubation, cells were treated with different concentrations (0.5 - 10 μM) of shikonin for 48 h. After 48 h in culture, the cells were then treated with 5 mg/ml MTT at 37°C for 4 h. The reduction product, MTT-formazan, was solubilized with Dimethyl sulfoxide (DMSO). Absorption at 490 nm of each sample solution was considered to represent the MTT-reducing activity of the cells.

### Oil Red O staining and cell quantification

After differentiation was induced, cells were stained with Oil Red O (0.2% Oil Red O in 60% isopropanol). Oil Red O staining was determined using a modified protocol described by Ramírez-Zacarías JL et al. [18]. The cells were washed twice with phosphate buffered saline (PBS), fixed with 10% formalin for 1 h, dried, and stained with Oil Red O for 10 min. The cells were washed with 70% ethanol and water and then dried. The lipid content of stained cells was visualized by microscopy (Olympus IX71, Tokyo, Japan). The stained lipid droplets were dissolved in isopropanol and quantified by measuring absorbance at 510 nm.

### Protein extraction and western blot analysis

For the Western blot analysis, cells were washed with ice-cold PBS, collected, and centrifuged. The harvested cells were sonicated for 5 seconds at 40 W. Cell lysates were incubated for 20 to 30 min on ice and then centrifuged at 13,000 rpm at 4°C for 10 min. The protein concentration of the supernatant was determined with the Bio-Rad Protein Assay Reagent (Bio-Rad Laboratories, Hercules, CA, USA) using bovine serum albumin as the standard. Total proteins (30 μg per lane) were separated by 10% SDS-polyacrylamide gel electrophoresis and transferred to polyvinylidenedifluoride (PVDF) membranes (Millipore, Billerica, MA, USA). The membranes were blocked for 2 h at room temperature with 0.1% Tween 20 (Amresco Inc., Solon, OH, USA) in Tris-buffered saline containing 5% skim milk. After overnight incubation at 4°C with primary antibodies, the membranes were incubated with a horseradish peroxidase-conjugated secondary antibody for 1 h at room temperature. Immunodetection was carried out with ECL detection reagent (Amersham Biosciences, Uppsala, Sweden). All figures showing the results of quantitative analysis include data from at least three independent experiments.

### RNA extraction and real-time quantitative RT-PCR

Total RNA was isolated from 3T3-L1 adipocytes using the RNase kit (Nucleospin, iNtRON Biotechnology) and used to synthesize cDNA for analysis by real-time reverse transcription-polymerase chain reaction (RT-PCR) (LightCycler® 480 System, Roche, Basel, Swiss). Quantitative real-time PCR was performed in a 20 μl reaction mixture. The cycle conditions were as follows: 95°C for 5 min, followed by 50 cycles involving denaturing at 95°C for 20 s, annealing at 55°C for 15 s, and extension at 72°C for 30 s. The primer sequences were as follows: PPARγ forward 5′-TCGCTGATGCACTGCCTATG-3′; PPARγ reverse 5′-GAGAGGTCCACAGAGCTGATT-3′; C/EBPα forward 5′-GACTTCAGCCCC TACCTGGA-3′; C/EBPα reverse 5′-GTAGTCGTCGGCGAAGAGGT-3′; aP2 forward 5′-AGGCTC ATAGCACCCTCCTGTG-3′; aP2 reverse 5′-CAGGTTCCCACAAAGGCATCAC-3′; LPL forward 5′-TGTAACAATCTGGGCTATGAGATCAAC-3′; LPL reverse 5′-TCTTGCCATCCTCAGTC CC-3′; ERK forward 5′-GCTCACCCTTACCTGGAACA-3′; and ERK reverse 5′-GGACCAGAT CCAAAAGGACA-3′.

### Statistical analysis

Group results were compared by an analysis of variance (ANOVA), followed by Duncan’s test using SPSS 18.0 software. Data are expressed as the mean ± standard error of the mean (SEM). P < 0.05 was considered significant.

## Results

### Shikonin inhibits differentiation of 3T3-L1 preadipocytes

**Figure 1:**
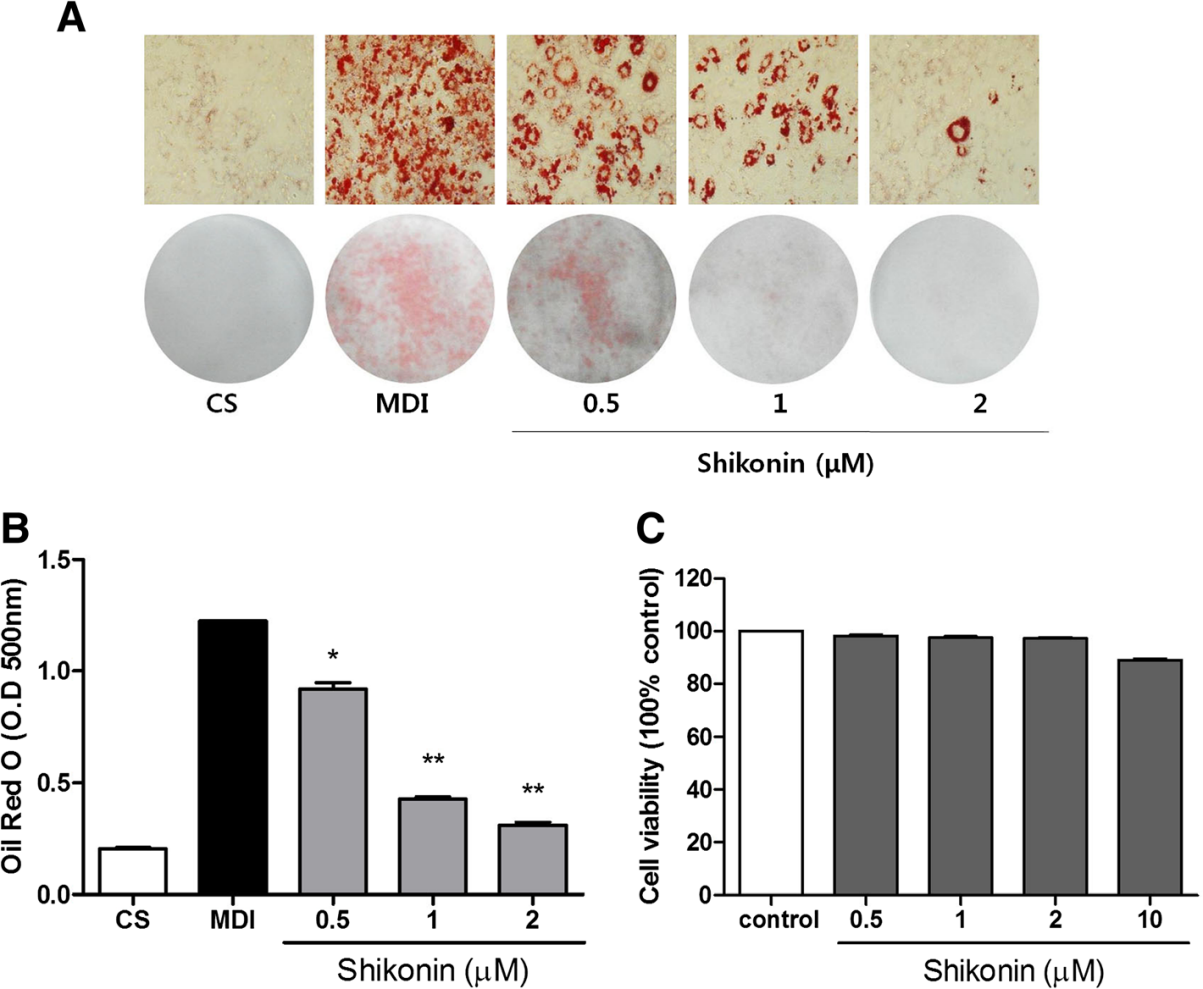
**Effect of shikonin on the lipid accumulation of 3T3-L1 adipocytes. (A)** Cell viability was determined by an MTT assay. **(B)** The lipid content was assessed by Oil Red O staining. **(C)** The absorbance of Oil Red O dissolved in isoprophanol was spectrophotometrically determined at 500 nm.

We performed an MTT assay to analyze the viability of 3T3-L1 preadipocyte cells treated with shikonin for 48 h. Shikonin did not show any effects on cell viability and cytotoxicity (Figure 1C).

To investigate the effects of shikonin on adipocyte differentiation, 3T3-L1 cells were induced to differentiate with MDI in the presence or absence of shikonin for 8 days. The effect of shikonin on the lipid accumulation of adipocytes was measured by Oil Red O staining. Shikonin inhibited the differentiation of 3T3-L1 pre-adipocytes in a dose-dependent manner (Figure 1A-B). Treatment with 0.5, 1 and 2 μM shikonin significantly decreased lipid droplets by 25.2%, 67.2% (P < 0.05), and 76.4% (P < 0.01), respectively, compared with MDI-treated cells. These results demonstrated that shikonin inhibited the differentiation of pre-adipocytes.

### Shikonin inhibits the expression of adipogenic transcription factors and genes

**Figure 2:**
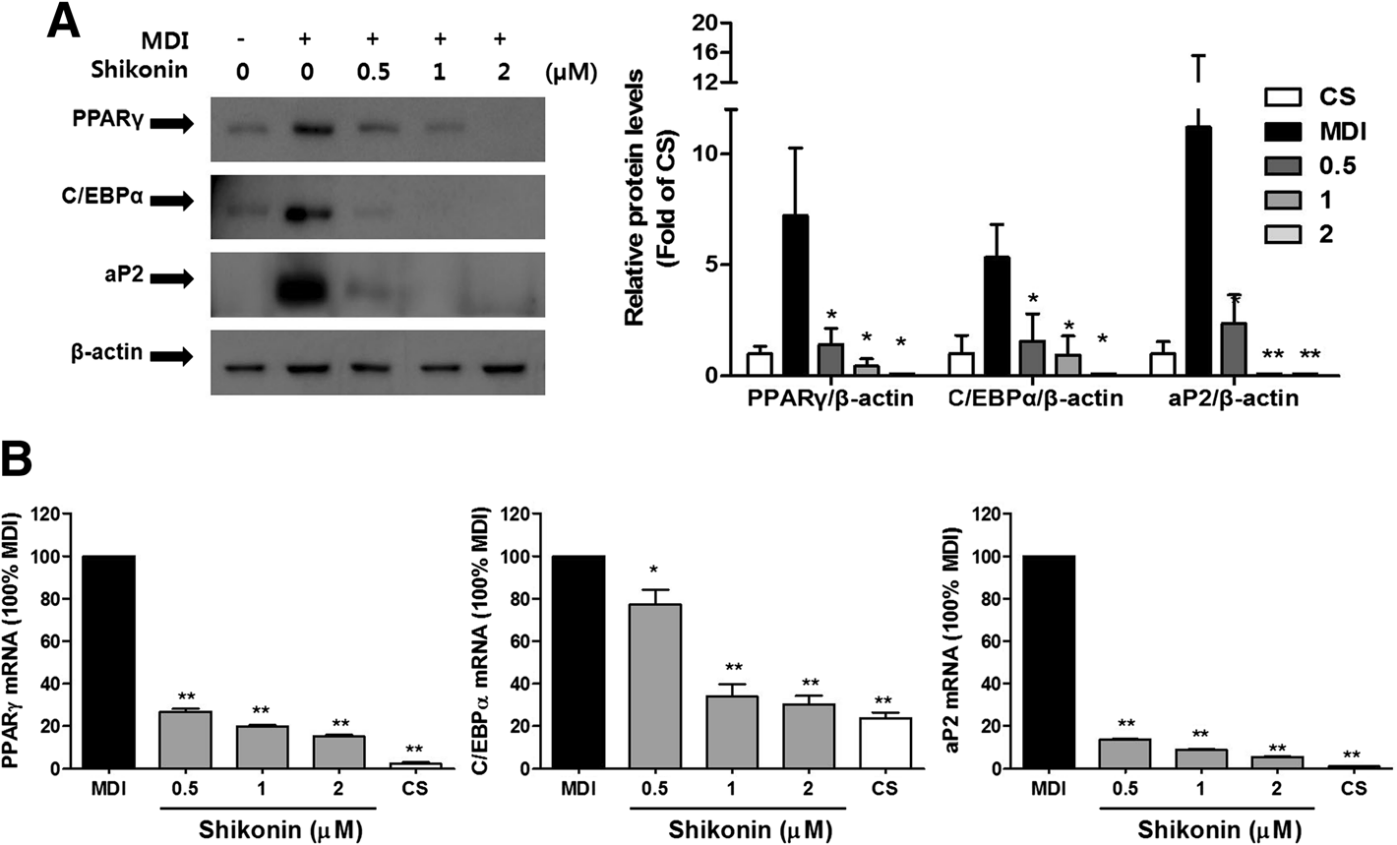
**Effect of shikonin on adipogenic transcription factor protein levels and gene expression in 3T3-L1 adipocytes. (A)** Western blot analysis was performed with antibodies against PPARγ, C/EBPα, aP2, and β-actin. **(B)** The expression of PPARγ, C/EBPα, and aP2mRNAs from 3T3-L1 adipocytes was measured by qRT-PCR.

Next, to examine whether shikonin inhibits adipocyte differentiation through the downregulation of adipogenic transcription factors and their target genes, we performed Western blotting and quantitative real-time PCR to analyze the protein and mRNA expression of PPARg, C/EBPa, and aP2. The protein levels of PPARγ, C/EBPα, and aP2 decreased with increasing dosages of shikonin in 3T3-L1 cells (Figure 2). Consistently, the mRNA expression of PPARγ, C/EBPα, and aP2 was also reduced by shikonin (Figure 2). These results demonstrate that shikonin inhibits adipogenesis through the downregulation of adipogenic transcription factors and their genes.

### Shikonin inhibits adipogenesis through the suppression of ERK 1/2 phosphorylation

**Figure 3:**
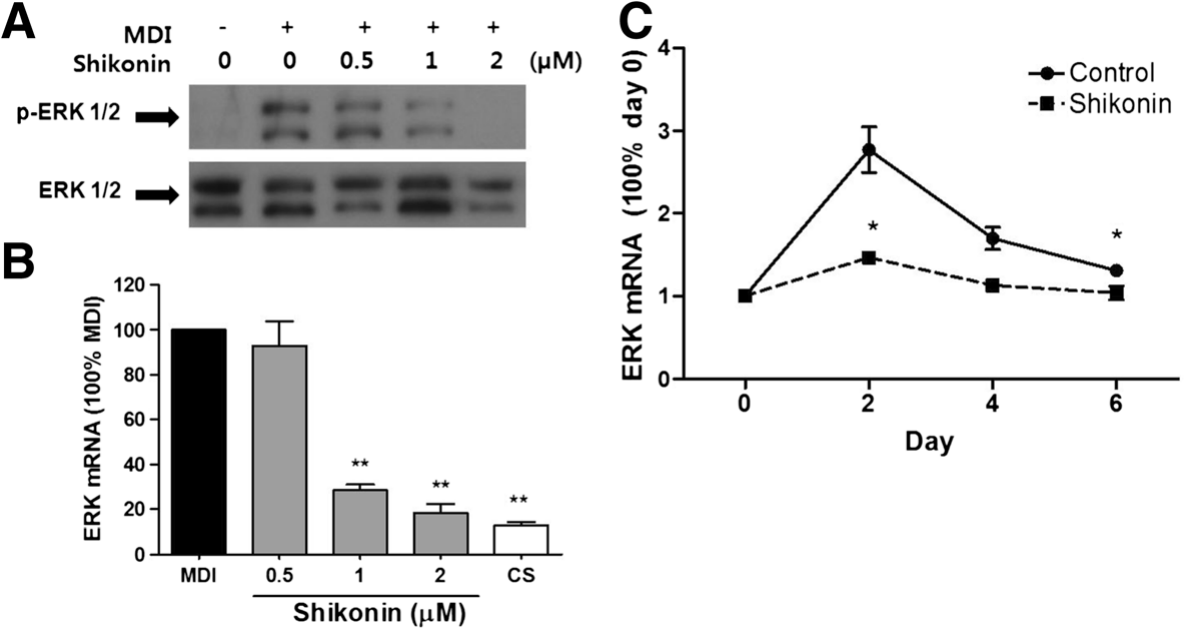
**Inhibitory effect of shikonin on ERK 1/2 phosphorylation in 3T3-L1 adipocytes. (A)** Protein level and **(B)** mRNA expression were determined with ERK 1/2. **(C)** Time-course of ERK expression as measured by qRT-PCR.

Many studies have suggested that MAPKs promote early-stage adipocyte differentiation by activating transcription factors [11,19-21]. The ERK1/2 signaling pathways has been reported to play a critical role for controlling adipogenesis [22]. To elucidate possible mechanisms underlying the inhibition of adipocyte differentiation by shikonin, we further examined whether regulation of ERK 1/2 phosphorylation is associated with the inhibition of adipocyte differentiation by shikonin (Figure 3A). Interestingly, shikonin markedly decreased the phosphorylation of ERK 1/2 in a dose-dependent manner. Additionally, mRNA expression of ERK 1/2 was inhibited by shikonin (Figure 3B). We also evaluated the effect of shikonin on ERK 1/2 mRNA expression at various time points during adipocyte differentiation. MDI-treated control cell showed significantly elevated ERK 1/2 phosphorylation between day 0 and day 2 compared with shikonin-treated cells. However, shikonin significantly downregulated ERK mRNA levels from day 4 to 6. These results suggest that shikonin inhibited adipocyte differentiation in the early stages.

Next, we sought to determine whether shikonin inhibits adipocyte differentiation via ERK 1/2 signaling pathway. 3T3-L1 cells were pretreated with ERK inhibitor PD98059 [23] or ERK activator FGF-2 [24] for 30 min, followed by induction of differentiation by MDI with or without shikonin (Figure 3B). Mature lipid accumulation and adipogenesis-related markers in matured adipocytes were determined on day 8 after treatment with MDI. As shown Figure 3B, pretreatment with PD98059 attenuated adipocyte differentiation. Shikonin also inhibited differentiation; the resulting adipocyte differentiationlevels were similar to those obtained with PD98059. Pretreatment with ERK activator FGF-2 increased the lipid droplet similar to MDI-treated control cells. However, shikonin decreased FGF-2–mediated activation of ERK 1/2 in 3T3-L1 cells.

PD98059 and shikonin consistently reduced the protein levels of adipogenic transcription factors (Figure 3C). In contrast, FGF-2 increased the protein levels of PPARγ, C/EBPα, and aP2. Additionally, co-treatment with shikonin and FGF-2 decreased the levels of these transcription factors compared with FGF-2-treated cells. Increased phosphorylation of ERK 1/2 was observed after FGF-2 stimulation in 3T3-L1 cells, whereas the phosphorylation levels were reduced by shikonin. Moreover, the inhibition of adipocyte differentiation was in accordance with the decrease in ERK1/2 phosphorylation; thus, inhibition of adipocyte differentiation by shikonin may be due to suppression of ERK 1/2 signaling. These results suggest that shikonin plays an important role in the inhibition of adipocyte differentiation via suppression of ERK 1/2 phosphorylation.

### Shikonin regulates ERK 1/2 phosphorylation in the early stages of adipogenesis

**Figure 4:**
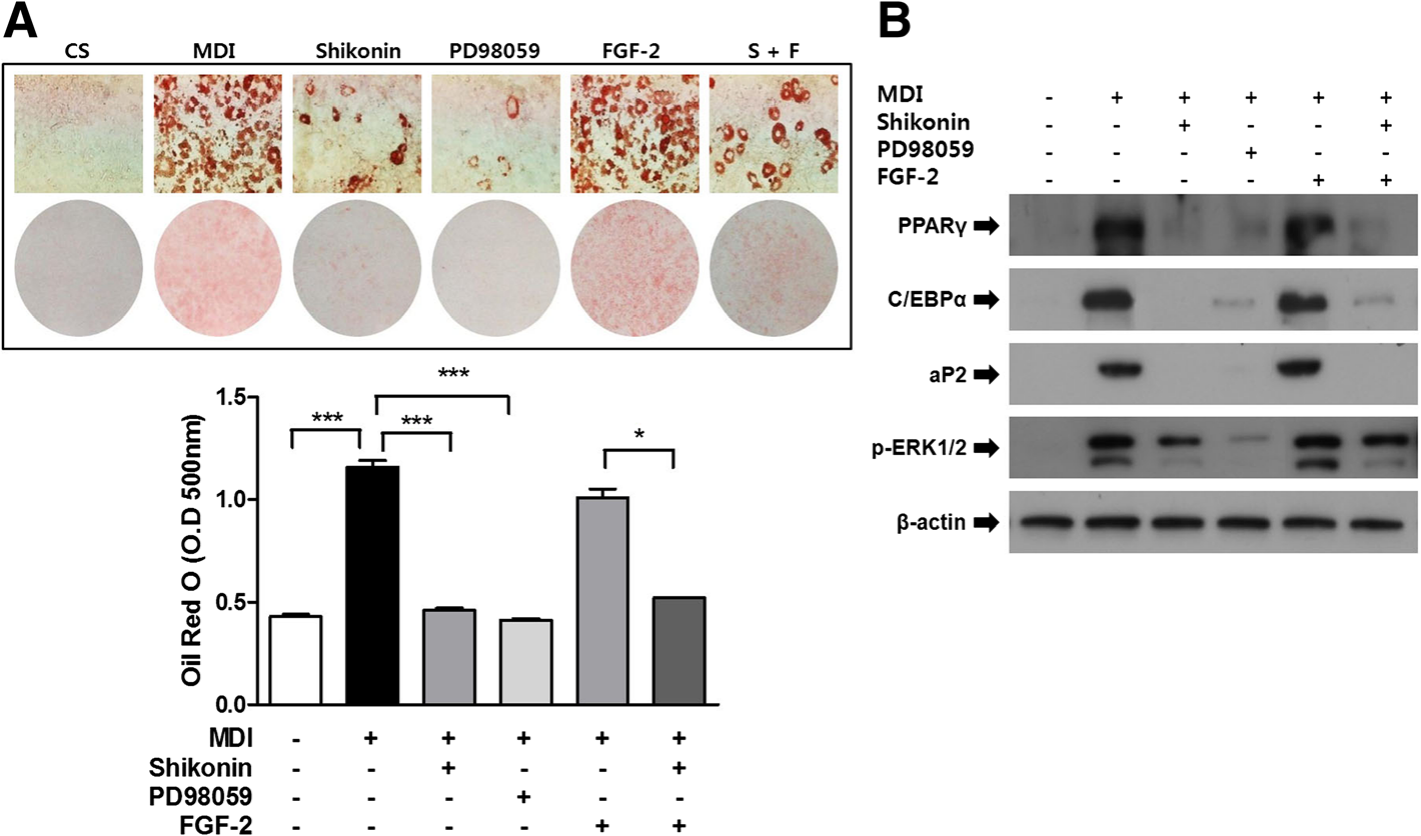
**Effects of the ERK inhibitor and activator on the inhibition of adipocyte differentiation by shikonin. (A)** The protein level and mRNA expression were determined with ERK 1/2. Cells were treated with **(A)** Shikonin (1 μM) and the ERK inhibitor PD98059 (10 μM) during adipocyte differentiation. After 8 days, the cells were stained with Oil Red O to determine the degree of lipid accumulation. The inhibitory effects of shikonin and PD98059 on ERK 1/2 signaling pathway were observed during adipocyte differentiation. **(B)** Western blot analysis performed with the ERK pathway and adipogenic marker genes.

ERK has been reported to promote differentiation in the early stages of adipogenesis in 3T3-L1 cells [9,25]. To confirm whether shikonin inhibits ERK phosphorylation in the early stages of adipogenesis, we examined time-course response of ERK 1/2 phosphorylation during the early differentiation period. Cells were pretreated with PD98059 (10 μM) or FGF-2 (1 nM) for 30 min prior to incubation with MDI in the presence or absence of 1 μM shikonin. ERK phosphorylation was determined at 5, 15, 30 min and 1 h after MDI treatment by Western blotting. As shown in Figure 4A, phosphorylation of ERK 1/2 was rapidly induced at 5 min after MDI treatment and maintained. FGF-2 also showed effects similar to MDI. ERK 1/2 phosphorylation was observed at 15 min after pretreatment of PD98059 and ceased after 1 h. ERK 1/2 phosphorylation was completely inhibited 30 min after treatment with shikonin. These results showed that shikonin suppressed at the early stage of adipogenesis after MDI treatment. To confirm the recovery effect of FGF-2 on ERK 1/2 phosphorylation inhibited by shikonin, cells were pretreated with 2 μM shikonin for 10 min and then various concentrations of FGF-2 for 30 min. As shown in Figure 4B, shikonin-mediated inhibition of ERK 1/2 phosphorylation was increased in a dose-dependent manner by FGF-2. These results showed that shikonin has an acute, direct effect on the ERK 1/2 signaling pathway through inhibition of ERK 1/2 phosphorylation.

## Discussion

Increased consumption of calorie-enriched foods with sugar and fats and a lack of physical activity lead to obesity. Obesity is a major risk for serious chronic diseases, including diabetes, cardiovascular disease, hypertension, and other health problems [1]. Adipocyte differentiation is an adaptive response to excess energy intake and induces obesity and metabolic diseases [8]. Accordingly, adipocytes are a therapeutic target for obesity, and many studies are being undertaken to prevent obesity through regulating adipogenesis. Many plants and phytochemicals have been reported to have biological activities without any side effects. Shikonin derivatives are a major compound of *Lithospermum erythrorhizon*, and shikonin has been reported to have antimicrobial, anti-inflammatory, and antitumor effects [12-14]. Shikonin and its derivatives, such as acetylshikonin and isobytyrylshikonin, have a similar structure and therefore have similar biological activity that acts through multiple mechanisms [13,26-29]. Here, we investigated the effect of shikonin on 3T3-L1 pre-adipocyte differentiation, focusing on the suppression of ERK 1/2 phosphorylation at the early stages of adipogenesis.

In the present study, shikonin significantly suppressed adipogenesis, which is characterized by increased lipid droplets in 3T3-L1 cells, and decreased the levels of adipogenic transcription factors, including PPARγ, C/EBPα, and the adipocyte-specific gene aP2.

Previous reports have shown that the MEK inhibitor, PD98059, significantly attenuates adipocyte differentiation and that FGF-2 induces the activation of the ERK 1/2 signaling pathway [30]. Based on these findings, the ERK 1/2 inhibitor, PD98059, and activator, FGF-2, were used to determine whether the anti-adipogenesis induced by shikonin is related to ERK 1/2 phosphorylation. Shikonin significantly inhibited ERK 1/2 phosphorylation and mRNA expression; PD98059 showed similar effects. As expected, FGF-2 treatment induced ERK 1/2 phosphorylation. We further confirmed that shikonin suppressed ERK 1/2 phosphorylation in the early stages of adipogenesis. These results are the first demonstration of the inhibition of ERK 1/2 signaling by shikonin.

The transcription factors PPARγ and C/EBPα have been demonstrated to play key roles in adipogenesis [2,3]. PPARγ, a member of the nuclear-receptor superfamily, is a master regulator of adipogenesis [31]. C/EBPα is required to maintain PPARγ expression and regulates insulin sensitivity in adipocytes [32]. Our results indicated that shikonin significantly suppressed lipid accumulation in dose-dependent manner via the decreased expression of PPARγ, C/EBPα, and aP2 (Figures 1, 2), which is consistent with the results of Yoon et al. [15].

aP2 is a member of the cytoplasmic fatty acid binding protein family, and its expression is highly regulated during the differentiation of adipocytes [33]. It is generally recognized that PPARγ and C/EBPα activate the downstream terminal adipocyte differentiation marker genes of aP2 [34].

Adipocyte differentiation involves complex cellular pathways and requires the sequential regulation of adipogenic and lipogenic genes [19]. The MAPK signaling pathways activate a variety of transcription factors involved in adipocyte growth and differentiation [35]. Previous studies have suggested that p38 has positive and negative effects on adipocyte differentiation [36,37]. Importantly, ERK 1/2 has been reported to play an essential role in cell proliferation and controlling adipogenesis [21]. ERK phosphorylation is necessary for the expression of the adipogenic transcriptional factors PPARγ and C/EBPα [24,38]. The present study showed that ERK 1/2 phosphorylation was suppressed by shikonin. Additionally, shikonin markedly reduced ERK 1/2 mRNA expression (Figure 3).

To confirm the more specific role of shikonin in the ERK signaling pathway, cells were treated with PD98059 or FGF-2. Pretreatment with PD98059 blocked ERK 1/2 phosphorylation and inhibited adipocyte differentiation (Figure 4). Similarly, shikonin also inhibited the phosphorylation of ERK 1/2, as well as the protein levels of adipogenic transcription factors. Furthermore, pretreatment with FGF-2 stimulated ERK 1/2 phosphorylation, and shikonin markedly attenuated the FGF-2-induced phosphorylation of ERK 1/2 (Figure 4B).

**Figure 5:**
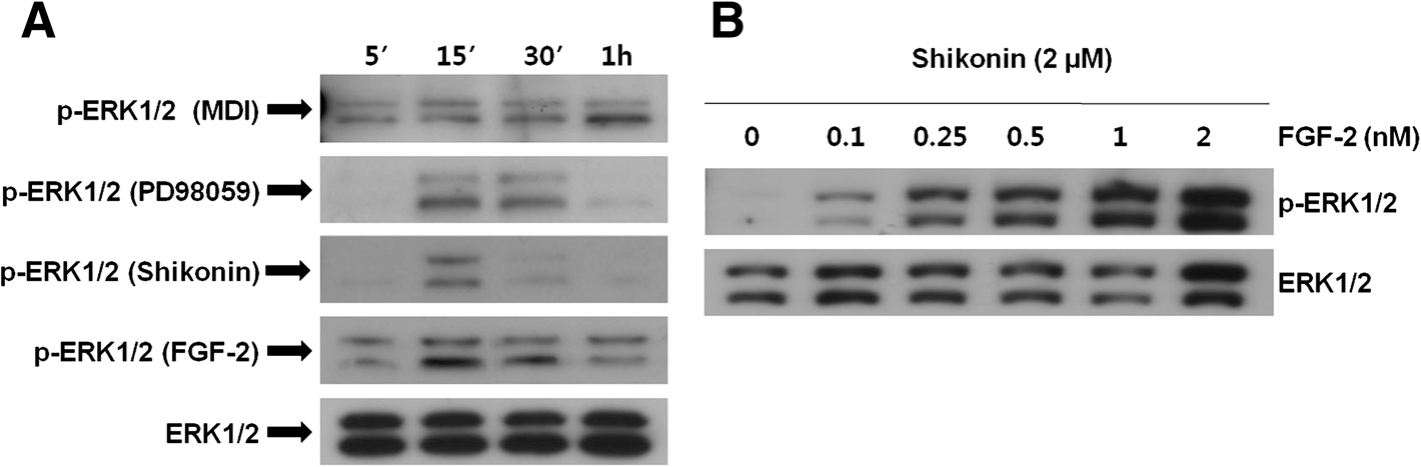
**Effect of shikonin on the ERK pathway during the early stages of adipogenesis. (A)** The time-dependent effect of PD98059 (10 μM), FGF-2 (1 nM), and shikonin (1 μM) on ERK 1/2 activity was analyzed by Western blotting. **(B)** The cells were treated with shikonin (2 μM) plus different doses of FGF-2. Cells were harvested at 10 min and examined with Western blotting as described in the Figure 2 legend.

Shikonin treatment inhibited ERK 1/2 phosphorylation in a time-dependent manner, which suggests that shikonin inhibits adipocyte differentiation by regulating ERK 1/2 phosphorylation in the early stages of adipogenesis (Figure 5A). To further confirm the inhibition of ERK 1/2 phosphorylation by shikonin, we investigated whether shikonin has a direct effect on ERK 1/2 phosphorylation. As expected, FGF-2 treatment inhibited shikonin-induced ERK 1/2 phosphorylation (Figure 5B). Taken together, these findings suggest that shikonin is able to block ERK phosphorylation at an early stage and inhibit the expression of adipogenic transcription factors by modulating the ERK-mediated signaling pathway during adipocyte differentiation. Further *in vivo* studies are necessary to determine the molecular mechanisms of shikonin-induced ERK 1/2 phosphorylation inhibition.

## Conclusions

Our results show that shikonin suppresses adipogenesis in 3T3-L1 cells by downregulating the expression of PPARγ and C/EBPα through the ERK signalling pathway at the early stages of adipogenesis. Therefore, these data indicate that shikonin is a potent and specific inhibitor of the ERK pathway in adipocyte differentiation and that shikonin may be useful agent in the prevention of obesity. Further studies are needed to elucidate the potential role of kinase inhibitors.

## Abbreviations

C/EBPα: CCAAT/enhancer binding protein α; PPARγ: Peroxisome proliferator-activated receptor γ; MAPK: Mitogen-activated protein kinases; LE: Lithospermum erythrorhizon; ERK 1/2: Extracellular signal-regulated kinases 1 and 2.

## Competing interests

The authors declare that they have no competing interests.

## Authors’ contributions

SYG performed the cell biology studies of cultured cells. JYA, CHJ, BKM, and TYH conceived the study and experimental design and interpreted the experimental results. All authors contributed to manuscript preparations and approved the final manuscript.

## Pre-publication history

The pre-publication history for this paper can be accessed here:

http://www.biomedcentral.com/1472-6882/13/207/prepub
